# Wearable Inertial Sensor-Based Detection of Exercise-Induced Mobility Adaptations in Older Women: A Randomized Controlled Trial

**DOI:** 10.3390/bios16090496

**Published:** 2026-09-05

**Authors:** Mauricio Barramuño-Medina, Pablo Valdés-Badilla, Pablo Aravena-Sagardia, Jordan Hernandez-Martínez, Edgar Vásquez-Carrasco, Wilson Pastén-Hidalgo, Cristian Sandoval-Vásquez, Germán Gálvez-García

**Affiliations:** 1 Kinesiology Program, Faculty of Health Sciences, Universidad Autónoma de Chile, Temuco 4780000, Chile; mauricio.barramuno@uautonoma.cl; 2 Department of Physical Activity Sciences, Faculty of Education Sciences, Universidad Católica del Maule, Talca 3460000, Chile; 3 Sport Trainer Program, Faculty of Life Sciences, Universidad Viña del Mar, Viña del Mar 2520000, Chile; 4 Physical Education Pedagogy, Faculty of Education, Universidad Autónoma de Chile, Temuco 4780000, Chile; pablo.aravena@uautonoma.cl; 5 Department of Physical Activity Sciences, Universidad de Los Lagos, Osorno 5290000, Chile; jordan.hernandez@ulagos.cl; 6 Department of Education, Faculty of Humanities, Universidad de la Serena, La Serena 1700000, Chile; 7 Occupational Therapy School, Faculty of Psychology, Universidad de Talca, Talca 3465548, Chile; edgar.vasquez@utalca.cl; 8 Centro de Investigación en Ciencias Cognitivas, Faculty of Psychology, Universidad de Talca, Talca 3465548, Chile; 9 VITALIS Longevity Center, Universidad de Talca, Talca 3465548, Chile; 10 Department of Kinesiology, Faculty of Health Sciences, Universidad de Atacama, Copiapó 1530000, Chile; wilson.pasten@uda.cl; 11 Escuela de Tecnología Médica, Facultad de Salud, Universidad Santo Tomás, Los Carreras 753, Osorno 5310431, Chile; 12 Departamento de Medicina Interna, Facultad de Medicina, Universidad de La Frontera, Temuco 4811230, Chile; 13 Department of Experimental Psychology, Psychobiology and Behavioral Sciences Methodology, Universidad de Salamanca, 37005 Salamanca, Spain; germangalvezgarcia@usal.es

**Keywords:** older people, wearable electronic devices, gait analysis, resistance training, fall risk

## Abstract

Wearable inertial sensors have become tools for objectively assessing mobility in older people. This study analyzed whether wearable inertial sensors could detect exercise-induced mobility changes and compared the effects of multicomponent training (MCT) and elastic band training (EBT) on mobility and physical function in older women. Forty-two participants were randomly allocated to either the MCT (*n* = 21) or EBT (*n* = 21) group, where 38 (EBT: *n* = 19; MCT: *n* = 19) completed the 16-week intervention. Outcomes included instrumented Timed Up-and-Go (iTUG) assessed with a wearable inertial sensor, the Senior Fitness Test, maximal isometric handgrip strength, conventional TUG, anthropometric measurements, health-related quality of life, and blood biomarkers. Data were analyzed using age-adjusted linear mixed-effects models. The iTUG showed shorter completion time (*p* < 0.001), reduced middle-turn duration (*p* = 0.004), and increased cadence (*p* = 0.007). Significant time effects were also observed for chair stand (*p* = 0.011), arm curl (*p* < 0.001), and conventional TUG (*p* = 0.009). No significant group × time interactions were detected. After adjustment for multiple comparisons, no significant changes were observed in anthropometric measures, health-related quality of life, or blood biomarkers. Wearable inertial sensors detected training-related mobility changes, and both exercise programs improved physical function and mobility without between-group differences.

## 1. Introduction

Older women are disproportionately affected by age-related declines in mobility and physical function. Compared with older men, they experience a higher prevalence of frailty, sarcopenia, falls, and mobility limitations, placing them at greater risk of disability, loss of independence, and institutionalization [1,2,3]. Mobility is a fundamental determinant of independence, overall health, and quality of life in older people [1,2,3,4]. Progressive declines in muscle strength, balance, gait performance, and functional capacity impair mobility and increase the risk of falls, disability, institutionalization, and mortality [1,2,3,4]. Falls remain one of the leading causes of injury and functional decline in later life, underscoring the need for effective interventions that preserve mobility and reduce fall risk [3,4]. Accordingly, maintaining and improving mobility has become a central objective of healthy aging strategies and geriatric care [1,2].

Given the importance of mobility for preserving independence in later life, regular physical activity is widely recognized as one of the most effective non-pharmacological strategies for attenuating age-related declines in physical function [2,4]. Exercise interventions have consistently been associated with improvements in muscle strength, balance, mobility, and reduced fall risk among older people [4,5,6]. Among the recommended exercise approaches, multicomponent training (MCT) has received particular attention because it simultaneously targets multiple determinants of functional independence, including muscular strength, balance, coordination, flexibility, and mobility [5,6]. Accordingly, intervention studies have reported significant gains in physical performance, postural stability, and functional capacity following MCT programs in older people [7,8,9]. Elastic band training (EBT) has also emerged as a practical and effective alternative for this population [10,11]. Compared with conventional resistance-training equipment, elastic bands are low-cost, portable, and easily implemented in both community- and home-based settings. Previous studies have shown that EBT improves muscular strength, balance, mobility, and overall functional performance in older people [10,11,12,13,14]. Moreover, accumulating evidence suggests that EBT can elicit functional adaptations comparable to those achieved with more complex exercise interventions while requiring fewer resources and minimal specialized equipment [12,13,14].

Older women represent a particularly important target population for mobility-focused interventions because they experience a greater burden of age-related declines in physical function, frailty, sarcopenia, and fall risk than their male counterparts [1,3,4]. From a preventive perspective, community-dwelling older women who remain functionally independent are also particularly relevant, as preserving mobility before overt functional limitations emerge may contribute to maintaining independence and reducing future mobility-related risk [1,4].

Both MCT and EBT have been shown to improve physical function, mobility, and other health-related outcomes in older women [7,8,9,10,11,12,13,14]. However, direct comparisons of the effectiveness of these two exercise approaches remain scarce, particularly among community-dwelling older women. As exercise interventions become increasingly adopted to preserve mobility and functional independence, sensitive assessment tools are essential for accurately quantifying their effects. Functional mobility is commonly evaluated using standardized performance-based tests, among which the Timed Up-and-Go (TUG) test is one of the most widely used for assessing mobility and fall risk in older people [15,16]. The TUG incorporates several fundamental functional tasks, including standing up, walking, turning, and sitting down. Although the total completion time provides a global measure of mobility, it does not capture the contribution of individual movement components to overall performance [16,17]. As a result, exercise-induced adaptations in gait, turning, or transitional movements may remain undetected when evaluation relies solely on the overall TUG completion time.

To overcome these limitations, advances in wearable sensor technology have expanded the possibilities for objective mobility assessment in older people [18,19,20]. Recent developments in wearable sensing and wireless technologies have further broadened their application in healthcare, enabling portable and objective monitoring of physiological and movement-related parameters beyond conventional laboratory-based assessments [18]. Wearable inertial sensors enable detailed quantification of gait and movement characteristics during functional tasks while remaining practical for use in both clinical and research settings [18,19,20]. Wearable sensor-based gait analysis has demonstrated the potential to objectively characterize clinically relevant alterations in locomotor performance [19], while waist-mounted IMU-based systems have shown reliability and validity for fall-risk assessment in community-dwelling older people [20]. Their validity and reliability for assessing mobility impairments, gait performance, and fall-related parameters in older people have been well established [21,22,23,24,25,26]. Moreover, these devices can detect subtle changes in movement patterns that may not be identified using conventional clinical assessments alone [21,22,23,24]. Among their most promising applications is in the instrumented TUG (iTUG), which combines the clinical utility of the traditional TUG with objective analysis of its individual movement phases [25,26]. By separately quantifying sit-to-stand, gait, turning, and stand-to-sit performance, the iTUG provides a more detailed characterization of functional mobility than total TUG completion time alone. Importantly, this phase-specific approach extends beyond the acquisition of additional mobility variables by revealing how individual components contribute to overall performance and potentially identifying subtle exercise-induced adaptations that may not be apparent from global clinical measures [17,25,26,27]. This enhanced resolution is particularly valuable in intervention studies, where improvements may occur in specific movement phases without substantially reducing overall task duration. Consequently, wearable sensor–based assessments can provide complementary insights into mobility adaptations associated with functional performance and fall risk.

Despite growing evidence supporting the benefits of exercise interventions and wearable technologies for mobility assessment, few randomized controlled trials have directly compared MCT and EBT while simultaneously evaluating the ability of wearable inertial sensors to detect training-induced mobility adaptations in community-dwelling older women. Accordingly, the primary aim of the present study was to analyze whether a wearable inertial sensor system could detect exercise-induced adaptations in functional mobility through phase-specific analysis of the iTUG. The secondary aimed to compare the effects of MCT and EBT on sensor-derived mobility outcomes and complementary measures of physical function, body composition, health-related quality of life, and blood biomarkers. Given the established validity and clinical utility of wearable inertial sensors for mobility assessment [18,19,20,21,22,23,24], it was hypothesized that the wearable inertial sensor system would detect exercise-induced changes in gait and mobility parameters, providing a more detailed characterization of functional adaptations than conventional TUG completion time alone. Based on previous evidence demonstrating the beneficial effects of MCT and EBT on functional performance and mobility in older people [7,8,9,10,11,12,13,14], it was further hypothesized that both interventions would improve physical function and mobility, with greater benefits expected following MCT.

## 2. Materials and Methods

### 2.1. Study Design

This study was designed as a two-arm, parallel-group randomized controlled trial with a 1:1 allocation ratio, conducted in accordance with the Consolidated Standards of Reporting Trials (CONSORT) guidelines [28] and registered at ClinicalTrials.gov (Identifier: NCT07561879). A single-blind design was adopted, with outcome assessors remaining blinded to group allocation throughout the study. Outcome measures were collected at baseline (PRE) and immediately after the intervention (POST). The intervention consisted of 32 supervised exercise sessions delivered over 16 weeks, with participants training twice weekly. Participants assigned to the MCT group attended sessions on Mondays and Wednesdays, whereas those allocated to the EBT group trained on Tuesdays and Thursdays. Each session lasted approximately 60 min. Randomization was performed using a computer-generated allocation sequence created with Randomized.org (https://www.randomizer.org/). Group assignment was carried out by an independent researcher who was not involved in participant recruitment, outcome assessment, or intervention delivery. All outcome assessments were conducted under standardized laboratory conditions by trained evaluators, following the standardized testing procedures and positions established for each test and battery, as described in the corresponding methodological references. Identical testing procedures were maintained at both time points. No adverse events, exercise-related injuries, or pain during either the assessment procedures or the intervention sessions were reported throughout the study.

### 2.2. Participants

A total of 38 community-dwelling older women were enrolled and completed the study. The mean age was 69.0 ± 6.2 years in the MCT group and 71.7 ± 4.5 years in the EBT group. Participants were recruited from a primary healthcare center serving older people in Temuco, Chile. Recruitment took place in March 2025, and all baseline, intervention, and post-intervention assessments were conducted between March and July 2025 at the same facility. At enrollment, all participants lived independently and were able to perform activities of daily living without assistance. Eligibility criteria included age ≥60 years, the ability to understand and follow simple verbal instructions, and functional independence, defined as a score >43 on the Chilean Preventive Medical Examination for Older People (EMPAM; Ministry of Health of Chile) [29]. Exclusion criteria comprised any disability limiting exercise participation or assessment, musculoskeletal injury, ongoing physical rehabilitation, or temporary or permanent medical conditions that contraindicated physical exercise. Attendance was recorded throughout the intervention, and adherence to the exercise program was monitored.

**Figure 1:**
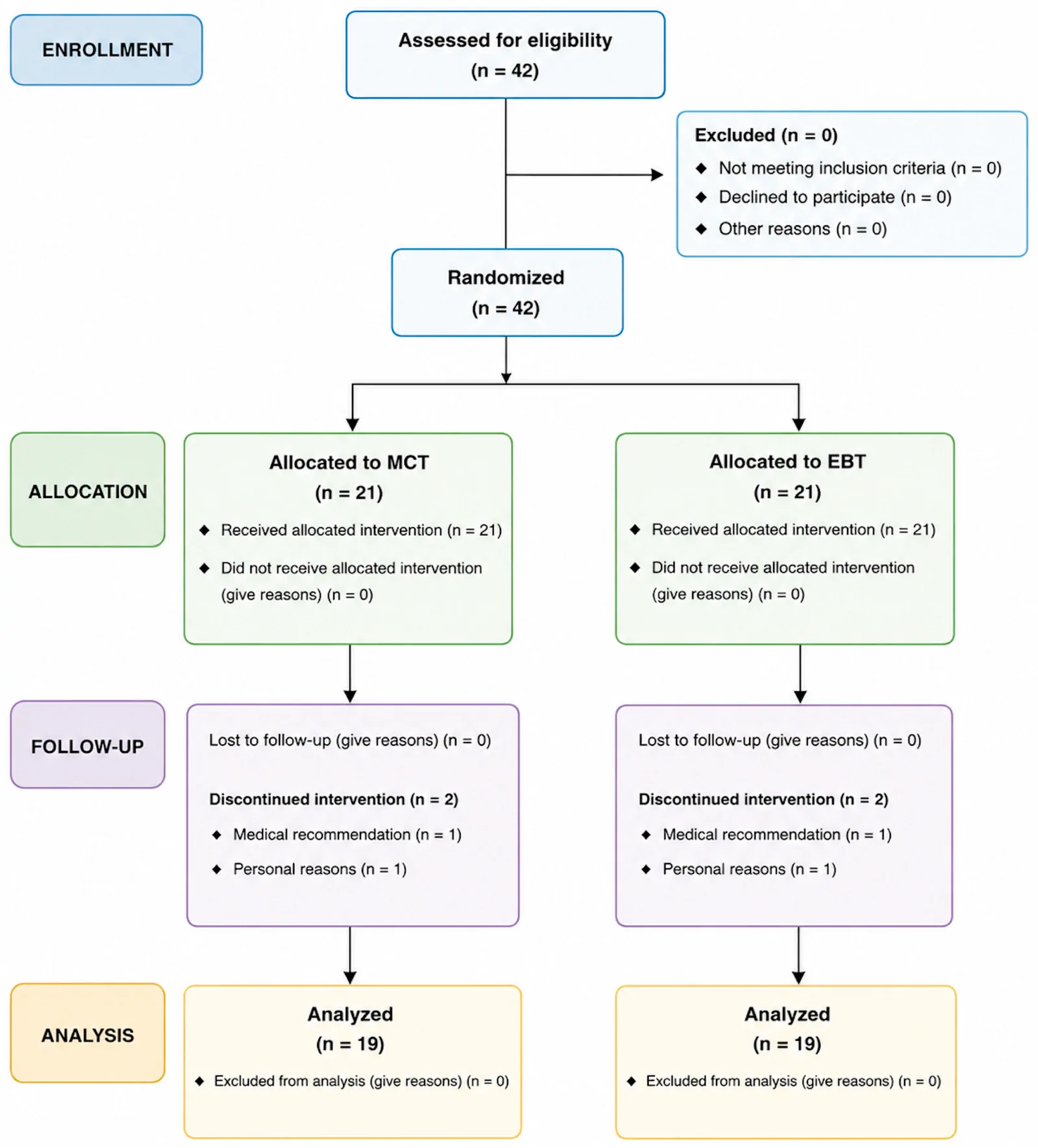
Flowchart of the recruitment process.

An *a priori* sample size calculation was performed using G*Power software (version 3.1.9.6; Franz Faul, University of Kiel, Kiel, Germany). The calculation was based on the time × group interaction effect for the TUG test reported by Hernandez-Martinez et al. [14] in a randomized controlled trial comparing EBT, MCT, and group-based dance in older women. The reported partial eta squared (ηp^2^ = 0.216) was converted to Cohen’s *f* (0.525), indicating a large effect size. Assuming a repeated-measures design with two groups and two assessment time points, a significance level of 0.05, and 80% statistical power, the minimum required sample size was estimated at 31 participants. To compensate for potential attrition, the recruitment target was increased to approximately 40 participants. A total of 42 participants were randomized, of whom 38 completed the intervention and were included in the final analyses (MCT: *n* = 19; EBT: *n* = 19). Four participants discontinued the intervention. The participant flow is presented in Figure 1.

Written informed consent was obtained from all participants before study enrollment. The study protocol was approved by the Research Ethics Committee of the Universidad Católica del Maule, Chile (Approval No. 29-2022), and all procedures were conducted in accordance with the ethical principles of the Declaration of Helsinki.

### 2.3. Primary Outcome: Instrumented Mobility

Instrumented mobility was assessed using the G-WALK^®^ wearable inertial measurement unit (BTS Bioengineering, Milan, Italy), which incorporates a triaxial accelerometer, triaxial gyroscope, and triaxial magnetometer operating at a sampling frequency of 100 Hz. Data acquisition and processing were performed using G-Studio software (version 3.3; BTS Bioengineering S.p.A., Milan, Italy). The sensor was secured over the fifth lumbar vertebra (L5) using an elastic belt in accordance with the manufacturer’s instructions. During the iTUG test, the system automatically quantified spatiotemporal and mobility-related variables, including total iTUG completion time, gait speed, cadence, sit-to-stand and stand-to-sit transition times, and turning performance. These outcomes were selected because they provide an objective characterization of mobility and may detect subtle changes associated with functional capacity and fall risk that are not captured by conventional clinical assessments alone [24,25]. Sensor data were processed automatically using the proprietary G-Studio software (version 3.3, BTS Bioengineering, Milan, Italy) according to the manufacturer’s predefined iTUG processing protocol, which automatically identifies the functional phases of the test and calculates the corresponding mobility parameters. No manual processing of the raw inertial signals was performed.

### 2.4. Complementary Outcomes

Complementary outcomes included conventional physical function, anthropometric measures, health-related quality of life, and blood biomarkers. Physical function was assessed using the Senior Fitness Test battery [30], which included the 30 s chair stand test to evaluate lower-limb functional strength, the arm curl test to assess upper-limb muscle endurance, the 2 min step test to measure aerobic endurance, the chair sit-and-reach test to evaluate lower-body flexibility, and the back scratch test to assess upper-body flexibility. Additional assessments included maximal isometric handgrip strength (MIHS), measured using a handheld dynamometer [31], and the conventional TUG test to evaluate functional mobility [32]. During the TUG, participants were instructed to rise from a standard chair, walk 3 m at their usual pace, turn around a marker, return to the chair, and sit down. Total completion time was recorded in seconds, with shorter times indicating better mobility performance.

Anthropometric assessment comprised body weight, height, body mass index (BMI), and waist circumference. Body weight was measured to the nearest 0.1 kg using a calibrated portable scale (Scale-Tronix 5002, Hillrom, Chicago, IL, USA), and height was measured to the nearest 0.1 cm using a telescopic stadiometer (Seca 220, Seca GmbH & Co. KG, Hamburg, Germany). BMI was calculated as body weight divided by height squared (kg/m^2^). Waist circumference was measured with a non-elastic anthropometric tape at the midpoint between the lower rib margin and the iliac crest according to the standardized protocols of the International Society for the Advancement of Kinanthropometry (ISAK) [33]. All anthropometric measurements were obtained with participants barefoot and wearing light clothing.

Health-related quality of life was assessed using the Medical Outcomes Study 36-Item Short-Form Health Survey (SF-36), a validated self-administered questionnaire that evaluates eight domains: physical functioning, role limitations due to physical health, bodily pain, general health, vitality, social functioning, role limitations due to emotional problems, and mental health. Scores for each domain range from 0 to 100, with higher scores indicating better perceived health status.

Blood biomarkers were obtained from fasting venous blood samples collected between 08:00 and 10:00 h after an overnight fast of at least 8 h. Blood collection was performed by trained healthcare personnel following standardized venipuncture procedures. Serum samples were analyzed in a certified clinical laboratory. Serum concentrations of fasting glucose, albumin, total cholesterol, high-density lipoprotein cholesterol (HDL-C), low-density lipoprotein cholesterol (LDL-C), and triglycerides were determined using automated standardized enzymatic and colorimetric methods.

### 2.5. Exercise Intervention

Both interventions (MCT and EBT) were implemented over 16 weeks, with participants attending two supervised sessions per week at the functional training facilities of the Sports Center of Universidad Autónoma de Chile. All sessions were supervised by a researcher with a master’s degree in Gerontology and experience in exercise interventions for older people. Before each session, participants underwent a brief clinical screening to confirm their readiness for exercise. Resting systolic and diastolic blood pressure and heart rate were measured using an automated blood pressure monitor (HEM-7120, OMRON Healthcare Co., Ltd., Kyoto, Japan) to ensure participant safety throughout the intervention.

Each training session lasted approximately 60 min and followed the same general structure in both groups. Sessions began with a 10 min warm-up consisting of joint mobility exercises and low-intensity aerobic activities, including treadmill walking, stationary cycling, or instructor-guided movement drills. The main training phase lasted approximately 40 min and comprised the exercises specific to the assigned intervention. Each session concluded with a 10 min cool-down involving dynamic and static stretching exercises to facilitate recovery and reduce residual muscle tension. The characteristics, progression, and exercise dosage of the MCT and EBT protocols are summarized in Table 1.

### 2.6. Multicomponent Training Program

The MCT intervention combined strength, balance, agility, and cardiorespiratory exercises within a circuit-based training format. Strength exercises targeted the major muscle groups of the upper (biceps, triceps, deltoids, and latissimus dorsi) and lower extremities (quadriceps, hamstrings, gluteals, and gastrocnemius). Additional exercises were incorporated to challenge static and dynamic balance, improve agility, and enhance cardiorespiratory fitness. Training was performed using body weight and a variety of exercise equipment, including dumbbells, kettlebells, medicine balls, and training poles. The training load was progressively increased throughout the intervention according to previously described periodization models [12,14,34]. During weeks 1–4, participants completed three sets of 10 repetitions per exercise with 2 min recovery intervals between sets. During weeks 5–8, the number of sets increased to four while repetitions and recovery intervals remained unchanged. In weeks 9–12, training volume was further increased to four sets of 12 repetitions. During the final phase (weeks 13–16), training volume was maintained, whereas recovery intervals were reduced to 90 s to increase the overall training stimulus. Exercise intensity was regulated using the OMNI-Resistance Exercise Scale [35,36], with participants exercising at ratings of perceived exertion between 5 and 8, corresponding to moderate-to-vigorous intensity. All repetitions were performed at a controlled tempo of approximately 2 s during the concentric phase and 4 s during the eccentric phase.

### 2.7. Elastic Band Training Program

**Figure 2:**
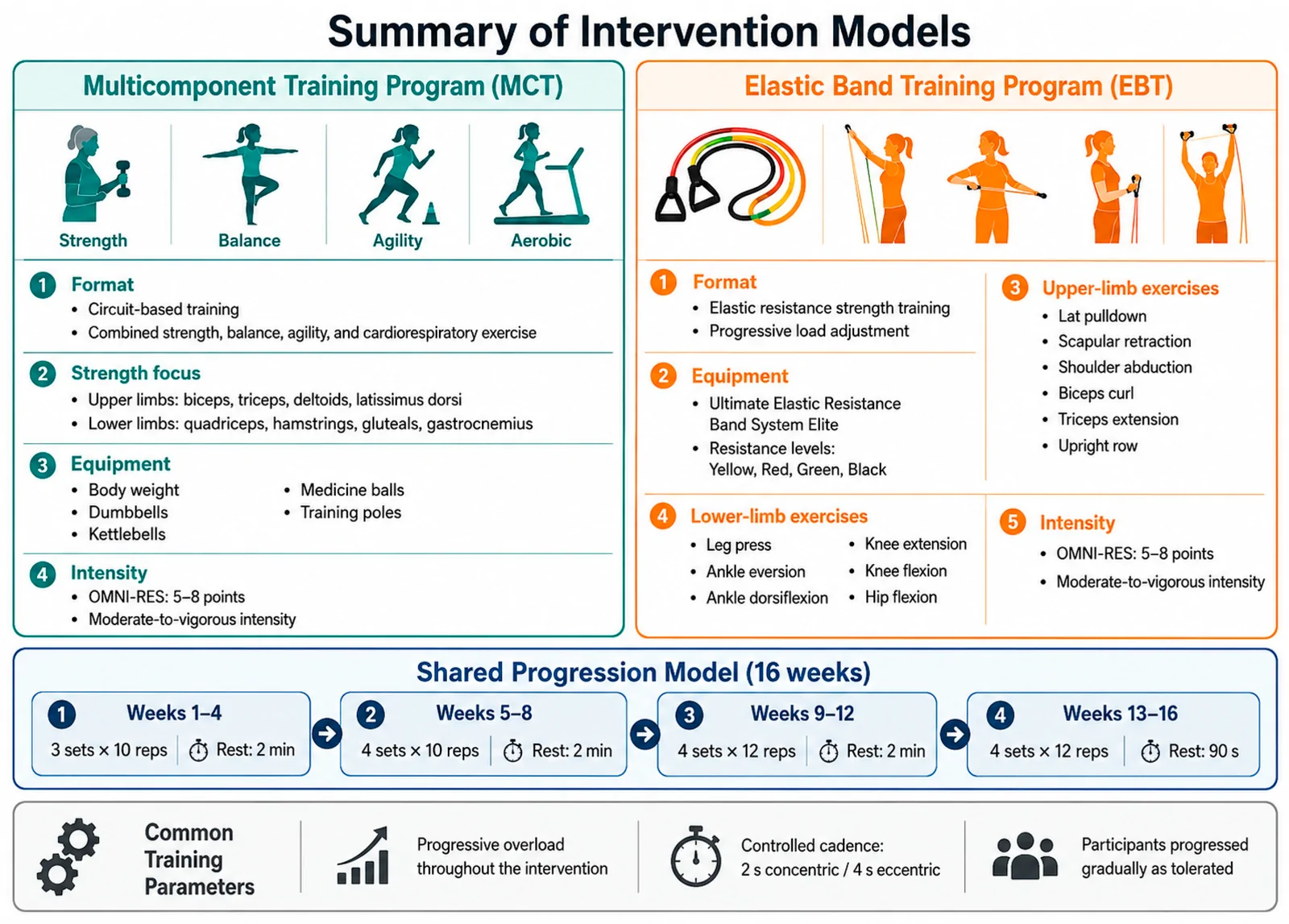
Overview of the multicomponent training (MCT) and elastic band training (EBT) intervention protocols.

The EBT protocol was based on previously published interventions that demonstrated its safety and effectiveness in older women [7,10,,^37^]. Resistance exercises were performed using the Ultimate Elastic Resistance Band System Elite (USA), which provides four color-coded resistance levels (yellow, red, green, and black) to enable progressive overload throughout the intervention. The program targeted the major muscle groups of the upper and lower extremities. Upper-extremity exercises included lateral pulldowns, scapular retractions, shoulder abductions, biceps curls, triceps extensions, and upright rows, whereas lower-extremity exercises comprised leg press movements, ankle eversion, ankle dorsiflexion, knee extension, knee flexion, and hip flexion. Participants began with the lowest resistance level and progressed to higher resistance bands as tolerated. The progression of training volume followed the same periodization model as the MCT intervention. Participants completed three sets of 10 repetitions during weeks 1–4, four sets of 10 repetitions during weeks 5–8, and four sets of 12 repetitions during weeks 9–12. During the final four weeks, training volume was maintained while recovery intervals were reduced from 2 min to 90 s to increase the training stimulus. Exercise intensity was monitored using the OMNI-Resistance Exercise Scale, with target ratings of perceived exertion between 5 and 8, corresponding to moderate-to-vigorous intensity. As in the MCT program, all exercises were performed at a controlled tempo of approximately 2 s during the concentric phase and 4 s during the eccentric phase. The main characteristics and shared progression model of both interventions are summarized in Figure 2.

### 2.8. Statistical Analysis

Statistical analyses were performed in R (version 4.5.1) using RStudio (version 2025.05.1+513). Linear mixed-effects models (LMMs) were fitted with the lme4 package [38] for all outcome variables, including anthropometric measures, physical function, instrumented mobility and gait, health-related quality of life, and blood biomarkers. Separate models were constructed for each outcome. Group (EBT vs. MCT), time (PRE vs. POST), and the group × time interaction were specified as fixed effects, whereas participant was included as a random intercept to account for repeated measurements. Age was included as a covariate in all models.

Fixed effects were evaluated using Type III analysis of variance with Satterthwaite’s approximation for the degrees of freedom. Estimated marginal means (EMMs) were calculated using the emmeans package, and pairwise comparisons were performed to assess within-group changes over time and between-group differences at each assessment time point. To account for multiple comparisons, the false discovery rate (FDR) procedure was applied separately within each outcome domain. Effect sizes were estimated using partial eta squared (η^2^p) and interpreted as small (0.01), medium (0.06), or large (0.14). Results are reported as F-values, degrees of freedom, *p*-values, FDR-adjusted *p*-values (pFDR), and effect sizes, as appropriate. Statistical significance was set at *p* < 0.05.

All analyses followed a per-protocol approach. Participants who discontinued the intervention before the post-intervention assessment (*n* = 4) were excluded from the analyses, and no imputation of missing outcome data was performed.

## 3. Results

A total of 38 participants completed the intervention and were included in the final analyses (EBT: *n* = 19; MCT: *n* = 19), and mean adherence was 90.5% in both groups. Baseline participant characteristics are presented in Table 2. Age and waist circumference did not differ significantly between groups, whereas participants in the EBT group had significantly higher body weight and BMI than those in the MCT group. Model-estimated PRE and POST values with 95% confidence intervals for all study outcomes are provided in Supplementary Table S1.

### 3.1. Instrumented Mobility Outcomes

**Figure 3:**
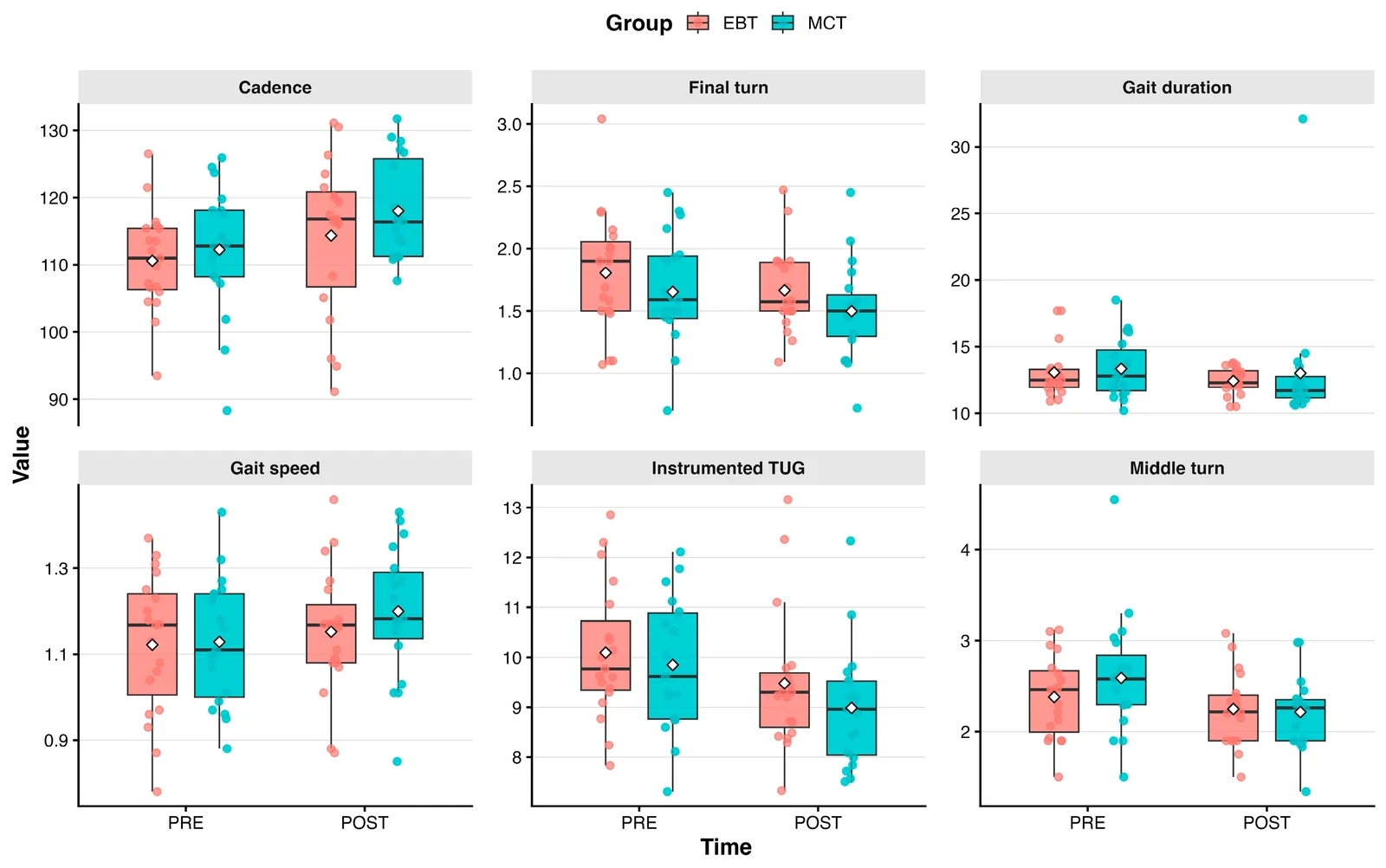
Changes in instrumented mobility and gait outcomes assessed using the G-Walk wearable inertial sensor system following the intervention period. Sensor-derived variables included cadence (steps/min), defined as the number of steps per minute during gait; final-turn duration (s), the time required to complete the turning maneuver upon returning to the chair and before sitting down; gait duration (s), the duration of the walking component of the iTUG; gait speed (m/s), the average walking speed during the gait component; instrumented TUG duration (s), the total time required to complete the iTUG; and middle-turn duration (s), the time required to complete the 180° change-of-direction turn at the 3 m marker before initiating the return walking phase. Boxplots represent the median and interquartile range, with individual participant values superimposed. White diamonds indicate the observed group mean. Measurements were obtained before (PRE) and after (POST) the intervention. EBT: Elastic band training; MCT: Multicomponent training; TUG: Timed Up-and-Go.

Instrumented mobility outcomes assessed using the G-WALK system are presented in Figure 3. Significant main effects of time were observed for iTUG performance (F_(1,38.00)_ = 12.70, *p* < 0.001, pFDR = 0.006, η^2^p = 0.251), middle-turn duration (F_(1, 38.00)_ = 9.36, *p* = 0.004, pFDR = 0.012, η^2^p = 0.198), and cadence (F_(1, 38.00)_ = 8.01, *p* = 0.007, pFDR = 0.015, η^2^p = 0.174).

Following the intervention, iTUG completion time decreased by 0.61 s (−6.1%) in the EBT group and by 0.87 s (−8.7%) in the MCT group. Middle-turn duration was reduced by 0.13 s (−5.6%) and 0.38 s (−14.4%) in the EBT and MCT groups, respectively. Cadence increased by 3.75 steps/min (+3.4%) in the EBT group and by 5.73 steps/min (+5.1%) in the MCT group. No significant main effects of group or group × time interactions were observed for any instrumented mobility outcome.

### 3.2. Physical Function Outcomes

**Figure 4:**
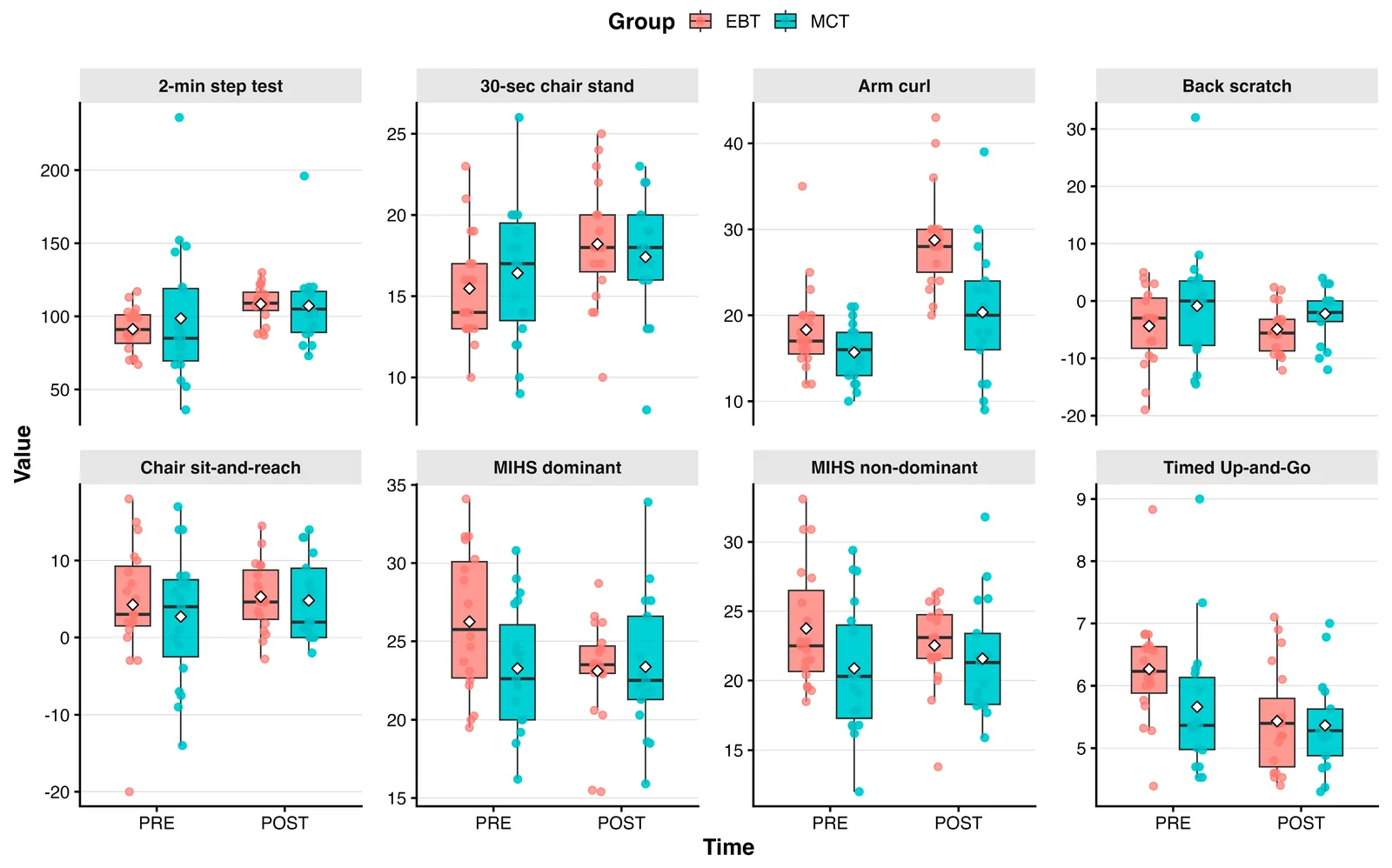
Changes in physical function outcomes following the intervention period. Boxplots represent the median and interquartile range, with individual participant values superimposed. White diamonds indicate the observed group mean. Measurements were obtained before (PRE) and after (POST) the intervention. EBT: Elastic band training; MIHS: Maximal isometric handgrip strength; MCT: Multicomponent training.

Physical function outcomes are presented in Figure 4. Significant main effects of time were observed for the 30 s chair stand test (F_(1, 37.02)_ = 7.14, *p* = 0.011, pFDR = 0.030, η^2^p = 0.162), TUG performance (F_(1, 38.80)_ = 7.64, *p* = 0.009, pFDR = 0.030, η^2^p = 0.165), and arm curl performance (F_(1, 37.90)_ = 50.20, *p* < 0.001, pFDR < 0.001, η^2^p = 0.570). A significant main effect of group was also found for arm curl performance (F_(1, 38.11)_ = 11.31, *p* = 0.002, pFDR = 0.014, η^2^p = 0.229).

Following the intervention, chair stand performance increased by 2.74 repetitions (+17.9%) in the EBT group and by 0.97 repetitions (+5.8%) in the MCT group. Arm curl performance improved by 10.4 repetitions (+57.7%) and 4.5 repetitions (+27.8%) in the EBT and MCT groups, respectively. Likewise, TUG completion time decreased by 0.83 s (−13.3%) in the EBT group and by 0.30 s (−5.3%) in the MCT group, indicating improved functional mobility. No significant group × time interactions were detected for any physical function outcome.

### 3.3. Anthropometric Outcomes

**Figure 5:**
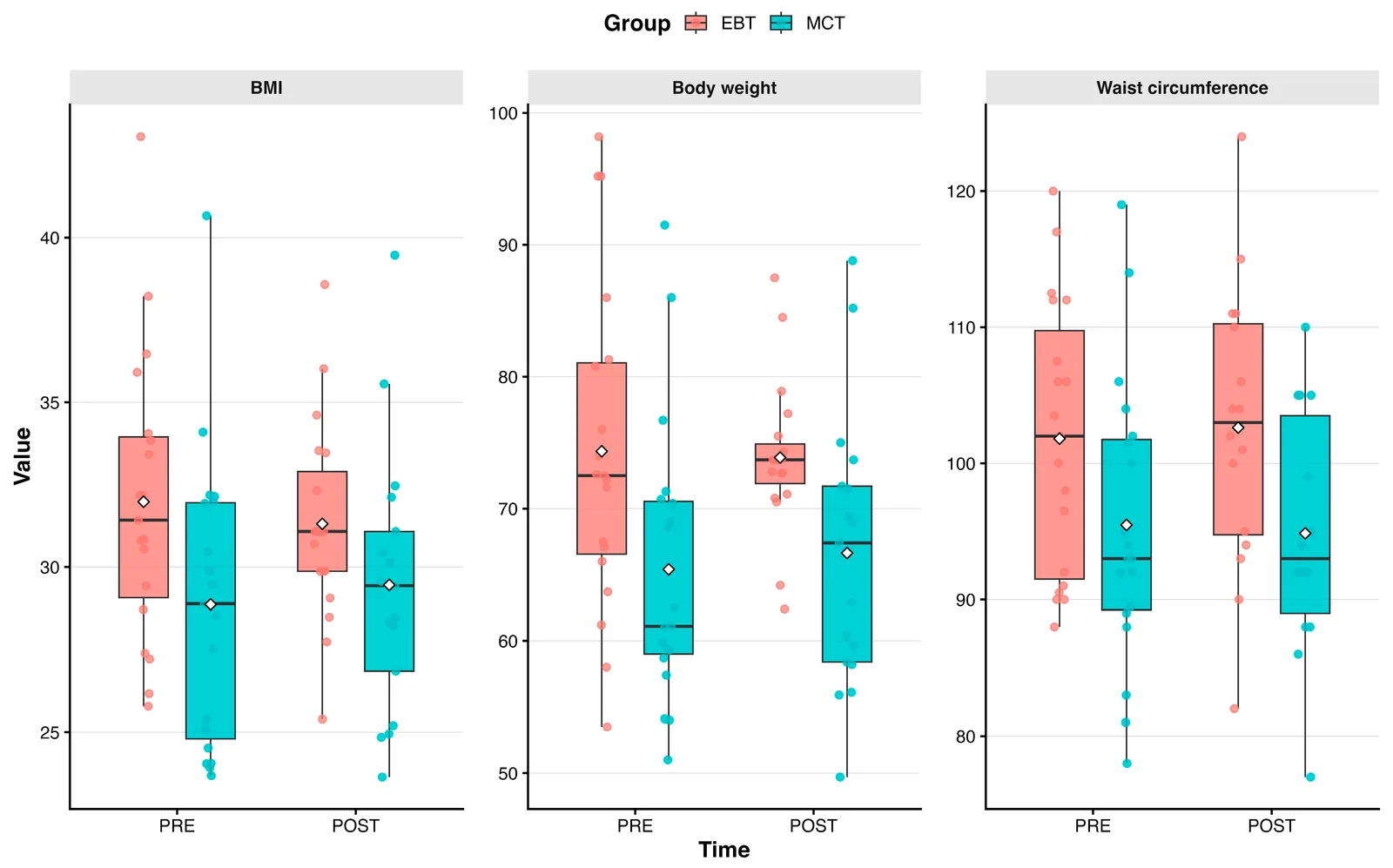
Changes in anthropometric outcomes following the intervention period. Boxplots represent the median and interquartile range, with individual participant values superimposed. White diamonds indicate the observed group mean. Measurements were obtained before (PRE) and after (POST) the intervention. BMI: Body mass index; EBT: Elastic band training; MCT: Multicomponent training.

Anthropometric outcomes are presented in Figure 5. Significant main effects of group were observed for body weight (F_(1, 37.77)_ = 9.48, *p* = 0.004, pFDR = 0.012, η^2^p = 0.20), BMI (F_(1, 37.40)_ = 5.42, *p* = 0.025, pFDR = 0.029, η^2^p = 0.13), and waist circumference (F_(1, 37.82)_ = 5.17, *p* = 0.029, pFDR = 0.029, η^2^p = 0.12). Body weight remained largely unchanged over the intervention, decreasing by 0.47 kg in the EBT group and increasing by 0.92 kg in the MCT group. Similarly, BMI showed minimal changes, decreasing by 0.56 kg/m^2^ in the EBT group and increasing by 0.23 kg/m^2^ in the MCT group. No significant main effects of time or group × time interactions were observed for any anthropometric outcome.

### 3.4. Health-Related Quality of Life Outcomes

**Figure 6:**
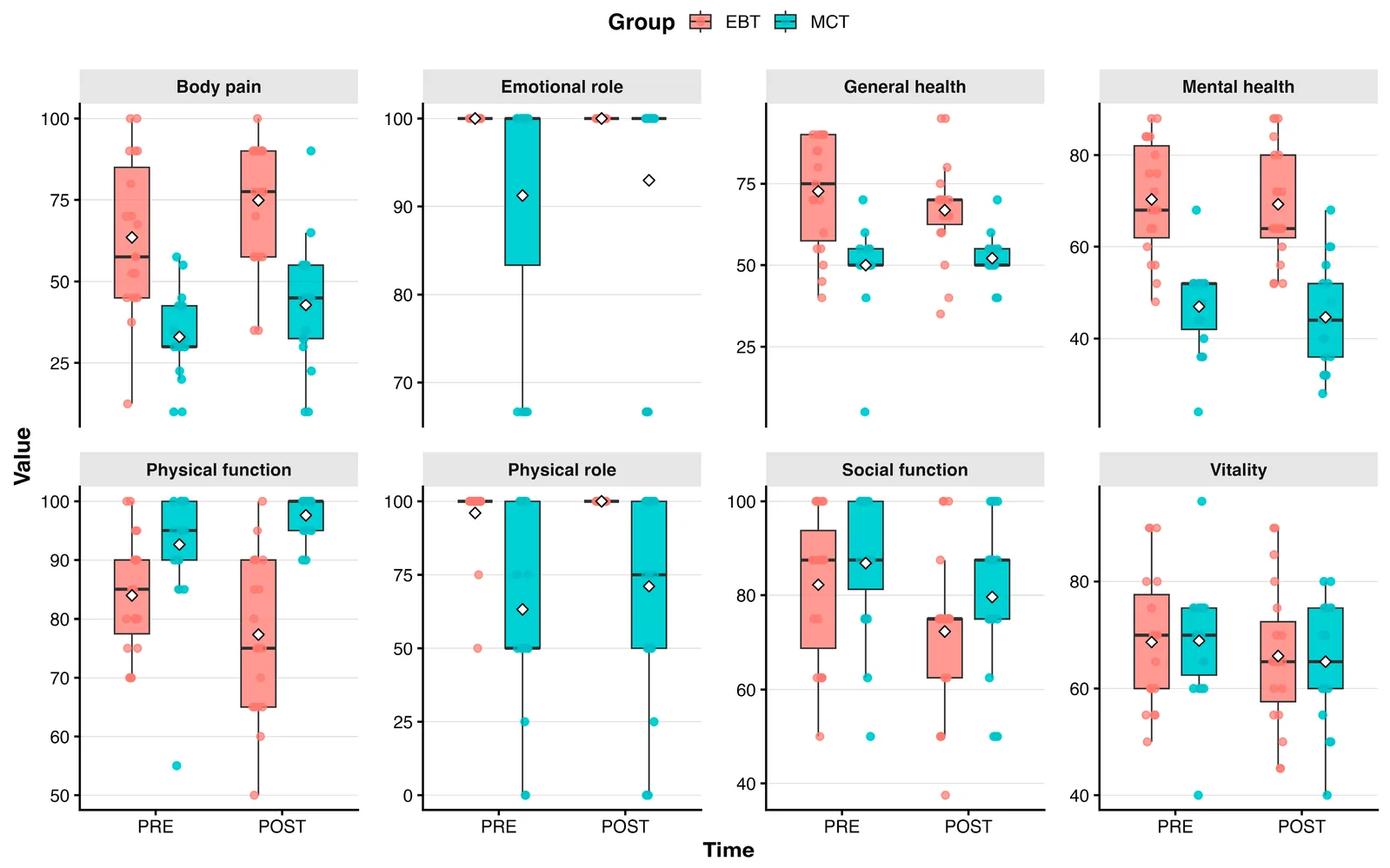
Changes in health-related quality of life outcomes following the intervention period. Boxplots represent the median and interquartile range, with individual participant values superimposed. White diamonds indicate the observed group mean. Measurements were obtained before (PRE) and after (POST) the intervention. Outcomes correspond to the SF-36 domains. EBT: Elastic band training; MCT: Multicomponent training.

Health-related quality of life outcomes are presented in Figure 6. Significant main effects of group were observed for physical functioning (F_(1, 76.00)_ = 44.26, *p* < 0.001, pFDR < 0.001, η^2^p = 0.368), bodily pain (F_(1, 38.00)_ = 37.61, *p* < 0.001, pFDR < 0.001, η^2^p = 0.497), role limitations due to physical health (F_(1, 38.00)_ = 20.96, *p* < 0.001, pFDR < 0.001, η^2^p = 0.355), role limitations due to emotional problems (F_(1, 38.00)_ = 7.70, *p* = 0.009, pFDR = 0.011, η^2^p = 0.168), mental health (F_(1, 38.00)_ = 60.02, *p* < 0.001, pFDR < 0.001, η^2^p = 0.612), and general health (F_(1, 38.00)_ = 30.49, *p* < 0.001, pFDR < 0.001, η^2^p = 0.445).

Over the intervention, bodily pain scores increased by 11.3 points (+18.1%) in the EBT group and 9.7 points (+28.6%) in the MCT group. Scores for role limitations due to physical health increased by 3.9 points (+4.1%) and 7.9 points (+12.4%) in the EBT and MCT groups, respectively. Physical functioning increased by 5.4 points (+5.8%) in the MCT group but decreased by 6.6 points (−7.9%) in the EBT group. Mental health scores decreased by 1.1 points (−1.5%) in the EBT group and by 2.3 points (−4.9%) in the MCT group. Likewise, vitality scores decreased by 2.6 points (−3.8%) in the EBT group and by 4.0 points (−5.7%) in the MCT group. However, no significant main effects of time or group × time interactions were identified for any health-related quality of life outcome after FDR correction.

### 3.5. Blood Biomarker Outcomes

No significant main effects of time or group, nor any group × time interactions, were observed for albumin, fasting glucose, total cholesterol, HDL-C, LDL-C, or triglycerides after FDR correction (all pFDR > 0.05).

## 4. Discussion

The primary objective of this randomized controlled trial was to compare the effects of MCT and EBT on physical function, mobility, anthropometric measures, health-related quality of life, and blood biomarkers in older women. A secondary objective was to evaluate whether a wearable inertial sensor system could detect training-induced changes in gait and mobility parameters associated with functional performance and fall risk. The findings partially supported the study hypotheses. Consistent with the first hypothesis, both interventions improved several indicators of physical function and mobility. However, contrary to expectations, MCT did not confer greater benefits than EBT, as no significant group × time interactions were observed for any outcome. In line with the second hypothesis, the wearable inertial sensor system detected significant improvements in instrumented mobility, including iTUG completion time, middle-turn duration, and cadence. Collectively, these findings indicate that both exercise modalities effectively enhance functional performance in older women, while wearable inertial sensors provide complementary information on mobility adaptations that may not be fully captured by conventional clinical assessments.

### 4.1. Mobility Adaptations and Wearable Sensor Findings

A key finding of the present study was that the wearable inertial sensor system detected significant improvements in instrumented mobility, including iTUG completion time, middle-turn duration, and cadence. These improvements occurred without significant group × time interactions, indicating that the pattern of change did not differ significantly between MCT and EBT. More importantly, the results demonstrate that wearable inertial sensors can objectively quantify changes in specific movement components that are not captured by conventional functional assessments alone. The absence of significant between-group differences should not be interpreted as a lack of responsiveness of the wearable assessment. Significant pre–post changes were detected in selected sensor-derived mobility outcomes across the intervention period, while there was no evidence that one exercise modality produced greater sensor-detected adaptations than the other. Rather, these findings indicate that the wearable system was able to capture changes in specific components of functional mobility within the intervention framework despite the absence of differential responses between MCT and EBT. In particular, the observed changes in middle-turn duration and cadence provide complementary information on mobility components related to dynamic balance, directional control, and locomotor performance, which are further discussed below.

Although improvements were also observed in the traditional TUG test, total completion time provides only a global measure of mobility and does not identify the movement components underlying performance changes. The TUG comprises multiple functional tasks, including sit-to-stand transitions, walking, turning, and stand-to-sit transitions, each of which depends on distinct combinations of balance control, lower-extremity strength, coordination, and locomotor function [15,16,17]. Consequently, similar reductions in overall TUG completion time may arise from different underlying movement adaptations. By independently quantifying each movement phase, the wearable sensor system offers a more comprehensive characterization of mobility performance and provides greater insight into the mechanisms through which exercise interventions influence functional mobility.

Among the instrumented mobility outcomes, the reduction in middle-turn duration warrants particular attention. Turning is widely regarded as one of the most demanding components of functional mobility in older people because it requires continuous regulation of the center of mass while maintaining dynamic stability [15,17]. Impaired turning performance has been associated with balance deficits, reduced mobility, and an increased risk of falls [17,18,19,20,25]. Therefore, the shorter middle-turn duration observed following training may reflect improvements in dynamic balance, movement coordination, and confidence during directional changes. These adaptations are clinically meaningful, as many falls occur during transitional movements rather than during steady-state walking.

Similarly, the increase in cadence may indicate a more efficient locomotor pattern after the intervention. Cadence is closely related to walking performance and functional mobility and has been identified as a sensitive marker of mobility decline in older people [18,19,20,21]. Although cadence should be interpreted within the broader context of gait performance, its increase alongside improvements in iTUG completion time is consistent with enhanced locomotor efficiency and functional mobility. Collectively, these findings suggest that both interventions promoted adaptations in mobility-related functions that are directly relevant to the performance of everyday activities.

The present findings further support the growing role of wearable inertial sensors as complementary tools for evaluating the effects of exercise interventions in older people. Although previous studies have established the validity and reliability of these systems for gait analysis and fall-risk assessment [18,19,20,21,22,23,39], relatively few randomized controlled trials have investigated their ability to detect exercise-induced mobility adaptations. In the present study, the wearable sensor system identified significant changes not only in overall mobility performance but also in specific movement phases, suggesting that instrumented assessments may be more sensitive than conventional clinical measures for detecting subtle functional adaptations. In this context, the contribution of wearable inertial sensing lies not merely in expanding the number of mobility outcomes available, but in enhancing the measurement resolution of functional assessment by objectively decomposing a global clinical task into clinically interpretable movement components. From a clinical perspective, this enhanced sensitivity may improve the monitoring of intervention effectiveness and provide a more comprehensive characterization of mobility changes associated with functional independence and fall risk.

### 4.2. Physical Function Adaptations

Both exercise interventions (MCT and EBT) elicited favorable improvements in functional performance, as demonstrated by enhanced chair stand, arm curl, and TUG performance. These findings are consistent with previous evidence showing that both training modalities improve muscle function and functional capacity in older people [5,6,7,8,9,10,11,12,13,14]. Improvements in chair stand and arm curl performance likely reflect enhanced lower- and upper-extremity muscle function, both of which are key determinants of independence and the ability to perform activities of daily living [1,2].

Contrary to the initial hypothesis, MCT did not produce greater functional gains than EBT. Although MCT is widely recommended because it simultaneously targets multiple physical capacities associated with healthy aging, including strength, balance, coordination, and mobility [5,6,7,8,9,40], the present findings indicate that a structured EBT program is equally effective in improving functional performance in community-dwelling older women. This observation is consistent with recent intervention studies reporting comparable functional benefits following EBT and more comprehensive exercise programs [12,13,14,41,42]. From a clinical and public health perspective, these findings are particularly relevant because elastic bands provide a low-cost, portable, and accessible training modality that can be implemented across a broad range of community and home-based settings.

The improvements observed in TUG performance further support the effectiveness of both interventions in enhancing functional mobility. As the TUG integrates lower-extremity strength, dynamic balance, coordination, and locomotor performance, improvements in test performance are likely to reflect integrated neuromuscular and functional adaptations rather than isolated changes in a single physical capacity [15,16].

### 4.3. Anthropometric Outcomes

Neither intervention produced significant changes in body weight, BMI, or waist circumference. Although exercise is widely recommended to improve body weight regulation and reduce central adiposity in older people, its effects on anthropometric outcomes are inconsistent, particularly in the absence of concomitant dietary interventions [9,12]. The lack of significant changes in the present study suggests that the exercise stimulus was sufficient to improve physical function and mobility but insufficient to elicit measurable alterations in body weight or central adiposity.

An alternative explanation is that neuromuscular adaptations often precede detectable changes in anthropometric measures. Early improvements in functional performance may reflect neuromuscular adaptations that can occur without concurrent reductions in body weight, BMI, or waist circumference [12,41]. In contrast, detectable changes in body composition and cardiometabolic outcomes may require a greater cumulative training stimulus and are influenced by factors such as training volume, intervention duration, and energy balance [9,12,42]. Accordingly, the absence of anthropometric changes should not be interpreted as a lack of intervention effectiveness, as both exercise programs produced meaningful improvements in functional performance and mobility.

### 4.4. Health-Related Quality of Life

Although significant main effects of group were observed for several SF-36 domains, no significant main effects of time or group × time interactions remained after FDR correction. These findings indicate that perceived health-related quality of life remained largely stable throughout the intervention period. The absence of significant intervention-related changes may, in part, reflect the relatively high baseline scores observed across several SF-36 domains, which could have limited the potential for further improvement because of ceiling effects. Previous systematic reviews have reported inconsistent effects of exercise interventions on health-related quality of life in older people, with the greatest benefits generally observed among individuals with poorer baseline health status, functional limitations, or a higher burden of chronic disease [43]. Therefore, the absence of significant changes in self-reported quality of life should not be interpreted as evidence of a lack of intervention effectiveness, particularly in light of the objective improvements observed in physical function and mobility.

### 4.5. Blood Biomarker Outcomes

Neither intervention produced significant changes in fasting glucose, total cholesterol, HDL-C, LDL-C, triglycerides, or albumin after adjustment for multiple comparisons. These findings are consistent with previous studies showing that exercise interventions of moderate duration and frequency do not consistently induce measurable cardiometabolic adaptations in relatively healthy older people [44,45]. Several factors may account for the absence of significant biomarker responses. First, participants exhibited no marked metabolic impairment at baseline, which may have limited the potential for physiological improvement. Second, cardiometabolic biomarkers are influenced by multiple factors beyond supervised exercise, including dietary intake, medication use, and habitual physical activity. Finally, longer intervention periods or combined exercise and nutritional strategies may be required to elicit clinically meaningful metabolic adaptations in this population. Together with the improvements observed in physical function and mobility, these findings suggest that functional adaptations may become apparent before measurable changes in circulating metabolic markers. This temporal dissociation is physiologically plausible because early training responses can be mediated by neuromuscular adaptations, whereas changes in systemic metabolic profiles may require a greater cumulative training stimulus or accompanying modifications in energy balance.

### 4.6. Strengths and Limitations

The present study has several strengths. First, it employed a randomized controlled design to directly compare two feasible and widely accessible exercise interventions for older women. Second, it provided a comprehensive evaluation of intervention effects by assessing physical function, instrumented mobility, anthropometric measures, health-related quality of life, and blood biomarkers. Most importantly, the incorporation of wearable inertial sensor technology enabled an objective and detailed assessment of mobility adaptations beyond those captured by conventional clinical tests.

Several limitations should be considered when interpreting the findings. First, the relatively small sample size may have reduced the statistical power to detect small-to-moderate between-group differences, increasing the possibility of type II error. Consequently, some clinically relevant effects may have remained undetected. Second, dietary intake and habitual physical activity outside the supervised training sessions were not monitored and therefore may have influenced anthropometric and biomarker outcomes. Third, training intensity was regulated in both groups using the same target range on the OMNI-Resistance Exercise Scale. Although this approach provided a common framework for individualizing and standardizing perceived internal effort across the two exercise modalities, it does not demonstrate that MCT and EBT elicited equivalent physiological training stimuli. Therefore, differences in the physiological demands of the two training formats cannot be excluded. Fourth, the absence of a non-exercise control group prevents the observed pre–post changes from being attributed exclusively to the training interventions. Finally, because the study included only community-dwelling older women, the findings may not be generalizable to older men or to populations with greater functional impairment.

### 4.7. Practical Implications

The findings of the present study underscore the potential value of wearable inertial sensors as complementary tools for mobility assessment in exercise and rehabilitation settings. Although conventional functional tests provide valuable information on overall mobility performance, wearable sensors enable objective quantification of specific movement components, such as turning performance and gait cadence, which may be particularly sensitive to exercise-induced adaptations in older people. This additional level of detail may assist clinicians, exercise professionals, and researchers in evaluating intervention effectiveness and individualizing exercise programs according to specific mobility profiles. Moreover, the portability, feasibility, and ease of implementation of wearable sensor systems support their integration into community-based exercise programs and primary healthcare settings, where objective monitoring of functional mobility may enhance fall-risk assessment and promote healthy aging. From a primary care perspective, phase-specific iTUG metrics may complement conventional screening by identifying mobility components that contribute to task performance but are obscured when only total TUG completion time is considered. Turning metrics may be particularly informative because turning requires dynamic balance, postural reorientation, and coordinated changes in direction, all of which are relevant to fall-related mobility. Thus, incorporating turning-phase and other segmented mobility indicators into routine assessment could help refine the identification of individuals with specific mobility deficits and support more targeted preventive interventions.

## 5. Conclusions

Wearable inertial sensor-based assessment identified favorable pre–post changes in specific components of functional mobility in community-dwelling older women, including iTUG completion time, middle-turn duration, and cadence. These findings highlight the value of the iTUG for objectively quantifying component-specific mobility adaptations beyond conventional global measures of functional mobility. Both MCT and EBT were also associated with favorable changes in selected functional outcomes, with no clear evidence of superiority of either intervention. Overall, wearable inertial sensing may provide a practical complementary approach for characterizing subtle mobility adaptations in older women, particularly those related to dynamic mobility and turning performance.

## Figures and Tables

**Table 1:** Characteristics of the central phase of multicomponent training and elastic band training interventions.

Component	MCT	EBT
Training approach	Circuit-based program combining strength, balance, agility, and cardiorespiratory exercises	Strength-training program using elastic bands
Training equipment	Body weight, dumbbells, kettlebells, medicine balls, and poles	Color-coded elastic bands (yellow, red, green, and black)
Main exercises	Squats, lunges, sit-to-stand, step-ups, rowing movements, overhead press, medicine-ball exercises.	Lat pulldown, scapular retraction, shoulder abduction, biceps curl, triceps extension, upright row, leg press, ankle eversion, ankle dorsiflexion, knee extension, knee flexion, and hip flexion
Additional components	Static and dynamic balance, agility, and aerobic exercises	Not included
Weeks 1–4	3 × 10 repetitions; 120 s rest	3 × 10 repetitions; 120 s rest
Weeks 5–8	4 × 10 repetitions; 120 s rest	4 × 10 repetitions; 120 s rest
Weeks 9–12	4 × 12 repetitions; 120 s rest	4 × 12 repetitions; 120 s rest
Weeks 13–16	4 × 12 repetitions; 90 s rest	4 × 12 repetitions; 90 s rest

EBT: Elastic band training; MCT: Multicomponent training. Both interventions lasted 16 weeks (two sessions/week; ~60 min/session) and included a 10 min warm-up, a 40 min main training phase, and a 10 min cool-down. Exercise intensity was maintained at 5–8 points on the OMNI-Resistance Exercise Scale (moderate-to-vigorous intensity). All exercises were performed using a controlled tempo of approximately 2 s during the concentric phase and 4 s during the eccentric phase.

**Table 2:** Baseline characteristics of participants according to intervention group.

Characteristic	MCT (*n* = 19)	EBT (*n* = 19)	*p*-Value
Age (years)	69.0 ± 6.2	71.7 ± 4.5	0.128
Body weight (kg)	65.4 ± 10.7	74.3 ± 12.6	0.024
BMI (kg/m^2^)	28.9 ± 4.4	32.0 ± 4.4	0.036
Waist circumference (cm)	95.5 ± 10.6	101.8 ± 10.0	0.066

Data are presented as mean ± standard deviation. EBT: elastic band training; MCT: multicomponent training; BMI, body mass index. Bold values indicate statistically significant between-group differences (*p* < 0.05).

## Data Availability

The datasets generated and/or analyzed during the current study are available upon reasonable request from the corresponding author.

## Supplementary Materials

The following supporting information can be downloaded at: https://www.mdpi.com/article/10.3390/bios16090496/s1, Table S1: Model-estimated pre- and post-intervention outcomes according to intervention group.

## Abbreviations

The following abbreviations are used in this manuscript:

MCT	Multicomponent training
EBT	Elastic band training
TUG	Timed Up-and-Go
iTUG	Instrumented Timed Up-and-Go
SF-36	36-Item Short-Form Health Survey
BMI	Body mass index
MIHS	Maximal isometric handgrip strength
LMM	Linear mixed-effects model
FDR	False discovery rate
