# Supplementary material for: Wearable Inertial Sensor-Based Detection of Exercise-Induced Mobility Adaptations in Older Women: A Randomized Controlled Trial
Source: Biosensors (Basel). 2026 Sep 5;16(9):496. doi: 10.3390/bios16090496 (PMC13604694; doi:10.3390/bios16090496)
Supplement: Supplementary file 1 [file biosensors-16-00496-s001.zip › biosensors-4471581-supplementary.pdf]

Table S1. Model-estimated pre- and post-intervention outcomes according to intervention group.

Outcome	MCT PRE, EMM (95% CI)	MCT POST, EMM (95% CI)	EBT PRE, EMM (95% CI)	EBT POST, EMM (95% CI)	Time pFDR	Group pFDR	Group × Time pFDR
Primary outcome: Instrumented mobility							
iTUG duration (s)	9.90 (9.28–10.51)	9.03 (8.41–9.65)	10.05 (9.43–10.67)	9.44 (8.82–10.05)	0.006*	0.556	0.837
Middle-turn duration (s)	2.61 (2.39–2.84)	2.24 (2.02–2.46)	2.36 (2.13–2.58)	2.22 (2.00–2.45)	0.012*	0.556	0.837
Final-turn duration (s)	1.67 (1.48–1.87)	1.52 (1.33–1.71)	1.78 (1.59–1.98)	1.64 (1.45–1.84)	0.129	0.556	0.943
Gait duration (s)	13.37 (12.05–14.69)	13.05 (11.73–14.36)	13.05 (11.73–14.36)	12.40 (11.09–13.72)	0.449	0.556	0.943
Gait speed (m/s)	1.13 (1.05–1.20)	1.20 (1.13–1.27)	1.13 (1.05–1.20)	1.16 (1.08–1.23)	0.129	0.607	0.837
Cadence (steps/min)	112.19 (107.90–116.48)	117.92 (113.63–122.21)	110.67 (106.38–114.96)	114.42 (110.13–118.71)	0.015*	0.556	0.837
Complementary outcomes: Physical function							
30-s chair stand (repetitions)	16.63 (14.89–18.38)	17.60 (15.78–19.43)	15.31 (13.58–17.05)	18.05 (16.32–19.79)	0.030*	0.738	0.339
Arm curl (repetitions)	16.04 (13.42–18.65)	20.50 (17.76–23.23)	18.05 (15.45–20.65)	28.48 (25.87–31.08)	<0.001*	0.014*	0.059
2-min step test (steps)	98.03 (84.68–111.39)	106.88 (92.84–120.91)	91.67 (78.39–104.95)	108.88 (95.60–122.16)	0.078	0.738	0.660
Chair sit-and-reach (cm)	2.44 (−0.74–5.63)	4.75 (1.41–8.10)	4.46 (1.30–7.63)	5.49 (2.33–8.66)	0.339	0.551	0.660
Back scratch (cm)	−0.88 (−4.49–2.73)	−0.63 (−4.32–3.05)	−4.36 (−7.94–−0.78)	−4.92 (−8.50–−1.34)	0.860	0.298	0.660
TUG (s)	5.68 (5.25–6.11)	5.38 (4.93–5.82)	6.25 (5.83–6.67)	5.42 (5.00–5.84)	0.030*	0.298	0.339
MIHS dominant (kg)	23.21 (21.29–25.12)	23.44 (21.44–25.45)	26.34 (24.39–28.30)	23.14 (21.24–25.04)	0.078	0.339	0.098
MIHS non-dominant (kg)	20.88 (18.95–22.82)	21.77 (19.75–23.78)	23.75 (21.82–25.68)	22.53 (20.60–24.46)	0.860	0.298	0.339
Anthropometric outcomes							
Body weight (kg)	65.06 (60.30–69.82)	65.98 (61.06–70.91)	74.60 (69.88–79.33)	74.13 (69.41–78.85)	0.889	0.012*	0.667
BMI (kg/m²)	28.78 (26.88–30.68)	29.04 (27.09–30.98)	32.04 (30.16–33.93)	31.37 (29.49–33.26)	0.889	0.029*	0.556
Waist circumference (cm)	95.38 (90.49–100.26)	92.66 (87.59–97.72)	101.89 (97.04–106.74)	100.94 (95.99–105.88)	0.164	0.029*	0.556
Health-related quality of life (SF-36)							
Physical functioning	93.02 (88.42–97.63)	98.02 (93.42–102.63)	83.56 (78.95–88.16)	76.98 (72.37–81.58)	0.720	<0.001*	0.091
Bodily pain	34.04 (25.35–42.73)	43.78 (35.09–52.47)	62.54 (53.85–71.23)	73.86 (65.17–82.54)	0.053	<0.001*	0.831

Outcome	MCT PRE, EMM (95% CI)	MCT POST, EMM (95% CI)	EBT PRE, EMM (95% CI)	EBT POST, EMM (95% CI)	Time pFDR	Group pFDR	Group × Time pFDR
Role limitations due to physical health	63.49 (52.01–74.97)	71.38 (59.90–82.86)	95.72 (84.24–107.20)	99.67 (88.19–111.15)	0.449	<0.001	0.831
Role limitations due to emotional problems	91.49 (86.72–96.25)	93.24 (88.48–98.01)	99.74 (94.97–104.50)	99.74 (94.97–104.50)	0.720	0.011*	0.831
Mental health	46.82 (41.46–52.17)	44.50 (39.15–49.85)	70.45 (65.09–75.80)	69.39 (64.04–74.75)	0.556	<0.001*	0.831
Social functioning	86.70 (79.24–94.17)	79.47 (72.00–86.93)	82.38 (74.91–89.84)	72.51 (65.04–79.97)	0.057	0.177	0.831
Vitality	68.87 (63.27–74.46)	64.92 (59.32–70.51)	68.77 (63.17–74.36)	66.13 (60.54–71.73)	0.449	0.837	0.831
General health	49.42 (43.27–55.58)	51.53 (45.37–57.68)	73.21 (67.06–79.36)	67.42 (61.27–73.57)	0.556	<0.001*	0.364
Blood biomarkers							
Albumin	3.80 (3.64–3.95)	3.67 (3.53–3.82)	3.64 (3.48–3.79)	3.63 (3.47–3.79)	0.644	0.504	0.879
Fasting glucose	114.25 (98.28–130.22)	104.49 (88.87–120.12)	108.94 (92.60–125.28)	114.54 (97.93–131.15)	0.851	0.820	0.259
Total cholesterol	195.81 (170.43–221.19)	184.95 (160.93–208.97)	175.42 (149.84–201.00)	168.00 (141.39–194.61)	0.644	0.504	0.947
HDL-C	60.32 (50.99–69.65)	59.30 (50.57–68.03)	58.30 (48.94–67.66)	56.55 (46.74–66.35)	0.859	0.762	0.947
LDL-C	104.61 (81.98–127.24)	97.15 (75.81–118.49)	95.44 (73.55–117.33)	87.04 (64.30–109.79)	0.644	0.666	0.947
Triglycerides	156.27 (107.93–204.61)	149.20 (102.54–195.86)	110.06 (60.89–159.23)	118.23 (67.75–168.70)	0.974	0.504	0.947

Note. Values are estimated marginal means (EMMs) with 95% confidence intervals derived from linear mixed-effects models adjusted for age. pFDR values correspond to the false discovery rate-adjusted p-values for the main effects of time and group and the group × time interaction. EBT: elastic band training; MCT: multicomponent training; iTUG: instrumented Timed Up-and-Go; TUG: Timed Up-and-Go; MIHS: maximal isometric handgrip strength; BMI: body mass index; HDL-C: high-density lipoprotein cholesterol; LDL-C: low-density lipoprotein cholesterol. * Indicates statistical significance ($pFDR < 0.05$).
